# Patterned FeNi soft magnetic strips film with tunable resonance frequency from 1 to 10.6 GHz

**DOI:** 10.1038/srep31773

**Published:** 2016-08-26

**Authors:** Yong Ren, Xinxi Li, Yan Wang, Jiankun Ren, Yan Zhang, Bo Dai, Haiyang Yan, Guangai Sun, Shuming Peng

**Affiliations:** 1 Institute of Nuclear Physics and Chemistry, China Academy of Engineering Physics, Mianyang, 621999, People’s Republic of China; 2 State Key Laboratory Cultivation Base for Nonmetal Composites and Functional Materials, Southwest University of Science and Technology, Mianyang, 621010, People’s Republic of China

## Abstract

Soft magnetic films with a wide-range tunable ferromagnetic resonance frequency are suitable for miniaturization and multifunctionalization of microwave integrated circuits. Fabrication of these films for high-frequency applications is usually complicated and difficult. We demonstrate a simple method to fabricate patterned FeNi soft magnetic strip films by magnetron sputtering and photolithography. Films prepared by this method exhibits a tunable in-plane uniaxial magnetic anisotropy (IPUMA) for different strip widths and gaps. As the strip widths changing from 500 to 2 *μ*m, the IPUMA field increases monotonically from 2.2 to 576 Oe and resonance frequency from 1 to 10.6 GHz(which covers four microwave bands, including the L,S,C and X bands) respectively. This ultra-wide-range adjustability of resonance frequency can be attributed to shape anisotropy of strips. Considering that FeNi alloy has relatively low magnetocrystalline anisotropy, so a wider adjustable range of resonance frequency could be obtained using materials with stronger magnetocrystalline anisotropy.

With rapid developments of wireless communications and high-frequency technologies in gigahertz range^1^, soft magnetic films materials have been widely studied in various fields such as radio-frequency^2^, electromagnetic compatibility devices^3^, electromagnetic interference problems and microwave absorption^4^,^5^,^6^. Resonance frequency (*f*_*r*_) is the most important factor which affects applications of these soft magnetic films. To obtain a higher *f*_*r*_ and its wider tunable range are the primary objectives for many studies^4^,^7^,^8^. Based on the Kittle equation 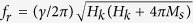, there are two factors to determine the *f*_*r*_ of the films, the saturation magnetization (*M*_*s*_) and anisotropy filed (*H*_*k*_) also called the in-plane uniaxial magnetic anisotropy (IPUMA) field^6^. In comparison with soft magnetic ferrites, magnetic metals have larger *M*_*s*_, thus various magnetic metals and alloys are used in high-frequency field^7^,^6^,^9^. Mainly, three anisotropies can be used to adjust the IPUMA of metal-soft-magnetic films. The first is the magnetocrystalline anisotropy. There are several methods to adjust this anisotropy, using different substrates^10^, oblique magnetron sputtering^8^,^11^, applying a magnetic field during deposition^12^ and magnetic field annealing^13^. All these methods require that the materials have strong magnetocrystalline anisotropy and sometimes right lattice constants. The second is the stress anisotropy of the film, which can be used to yield tunable *f*_*r*_ from 6.3 to 12.96 GHz^7^,^14^. The third is the shape anisotropy, such as patterned films with different strips^6^,^10^,^15^,^16^,^17^,^18^,^19^. The method works for materials either with or without strong magnetocrystalline anisotropy or stress anisotropy. Thus, the shape anisotropy is one of the most effective and versatile methods to tune the IPUMA of metal soft magnetic films. The shape anisotropy can usually be realized as the patterned strip films.

Patterned films with narrow strip arrays in a periodic structure that are used to control the IPUMA have been studied by many groups. Perrin *et al*. shifted *f*_*r*_ from 1 to 3 GHz for the amorphous CoZrNb and CoFeMoMnSiB strip patterned films which are fabricated by magnetron sputtering on a Kapton substrate^16^. Zhuang *et al*. obtained resonance frequency range of 1 to 5.3 GHz for the micropatterned FeNi films prepared by electroplating and lithography^17^. Han *et al*. studied the magnetization reversal mechanism of FeTa patterned strip films in 2013^6^. All previous studies indicate that shape anisotropy plays a key role in changing the resonance frequency of the soft magnetic patterned films. It is well known that the FeNi alloy has very low magnetocrystalline anisotropy^17^, thus it is an ideal system to adjust the IPUMA only by shape anisotropy. We studied micropatterned strip FeNi films (*Ni*_8_*Fe*_2_) fabricated by photolithography and magnetron sputtering with different shape anisotropies. An ultra-large range from 1 to 10.6 GHz of *f*_*r*_ were achieved in this work.

## Results

### Micrographs, thickness and roughness of the FeNi strip array film

**Figure 1:**
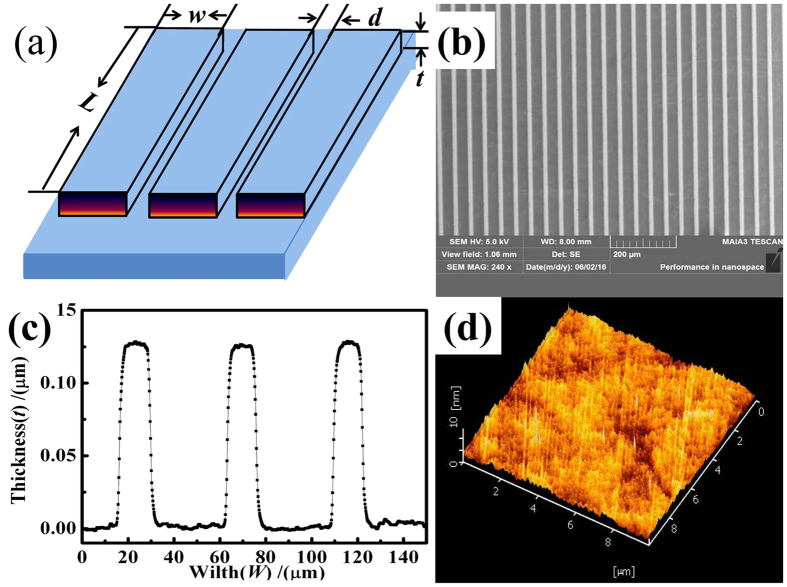
Diagram (**a**), SEM (**b**), Step profiler image (**c**) of the FeNi strip arrays, and AFM image (**d**) of the FeNi film.

Different strips were fabricated to yield different shape anisotropies. The diagram and a typical scanning electron microscopy (SEM) image of the strip arrays are shown in Fig. 1a,b, respectively. The length(L) and thickness(t) of the FeNi strips are fixed as 5000 *μm* and 120 nm repectively. The strip width (*W*) and gap size (*d*) range from 2 to 500 *μm* to obtain different demagnetization fields. Detailed data sets are provided in Tables 1 and 2. There are two sample sets. For the frist one, *d* is fixed while *W* varies. For the second one, *d* and *W* vary simultaneously with a constraint *d* = *W*. The SEM micrograph shows that strips are parallel and no one is broken.

Several instruments such as a step profiler, SEM and TEM were used to measure the film thickness of nanometers. A step profiler can measure the film thickness when the film has clear steps. SEM mainly observes the sample surface, so the film thickness can be measured when the film cross section is prepared. TEM can be used to study very thin films. It is difficult to obtain the exact thickness and roughness of the single layers buried in a multilayer film by conventional methods without serious damages. Fortunately, neutrons can penetrate deep into the matter, which makes it a unique probe for investigating bulk materials. We firstly investigated the total thickness of prepared Ta/FeNi/Ta via a step profiler. Fig. 1c shows a typical measurement result of the step profile for the FeNi strip film. The strip *W* and *d* are 15 and 30 *μm* respectively, and the total thickness is 130 nm. The results are consistent with the original design(Ta/FeNi/Ta:5 nm/120 nm/5 nm).

**Figure 2:**
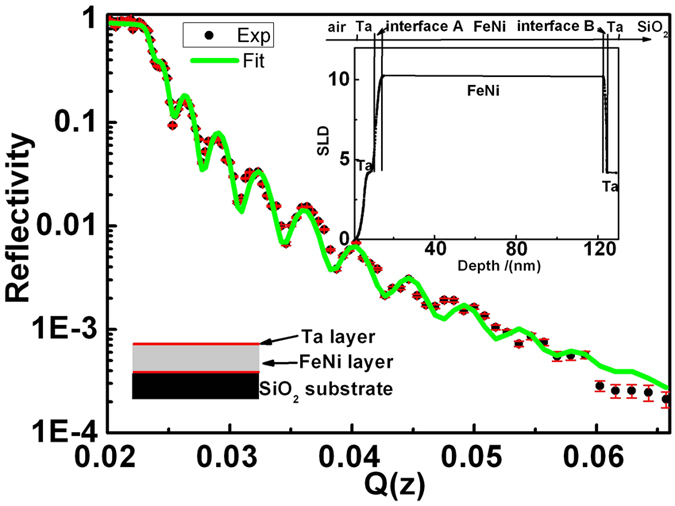
Neutron reflectivity spectra and scattering length density (inset) of the Ta/FeNi/Ta film.

Figure 1d shows the atomic force microscopy (AFM)image of the Ta/FeNi/Ta film, its surface undulation is about 2 nm based on a linear sweep model, whereas the root mean square roughness is 0.9 nm. The inner layer thickness and roughness of the Ta/FeNi/Ta multilayer film were studied by neutron reflectrometer at the China Mianyang Research Reactor (CMRR). Figure 2 shows a typical neutron reflectivity (R)–scatter vector (Q) curve and scattering length density (SLD, inset figure) of the Ta/FeNi/Ta film. The experimental data, error bar and fitting result are shown as the black dots, green sold line and red line, respectively. R decreased rapidly with Q. Nice neutron peaks indicate that the sample is well prepared. The SLD profile can be obtained by fitting the reflectivity data. As the neutrons see there are three layers of Ta/FeNi/Ta on the silicon substrate with thicknesses of 4, 118 and 4 nm respectively. The SLD profile also shows that the upper surface roughness of the FeNi film(interface A in Fig. 2) is about 2 nm, whereas that of the lower surface is about 1 nm (interface B in Fig. 2). The main reason for this phenomenon is due to accumulation of the surface roughness. The magnetic moment is parallel to the plane of the magnetic film for strong shape anisotropy cases, thus the magnetic moments near the surface will be difficult to rotate due to the surface roughness.

### FeNi film with various strip widths (*W*)

#### Static magnetic properties of the patterned FeNi strip film

To obtain different shape factor (*N*) of the magnetic strips, we changed the strip widths *W* as gap size *d* fixed to 30 *μm*. Based on the magnetism theory, *N* depends on the ratio between length (*L*) and *W*. Here, the length is always 5000 *μm*, and *W* = 500, 150, 60, 30, and 15 *μm*.

**Figure 3:**
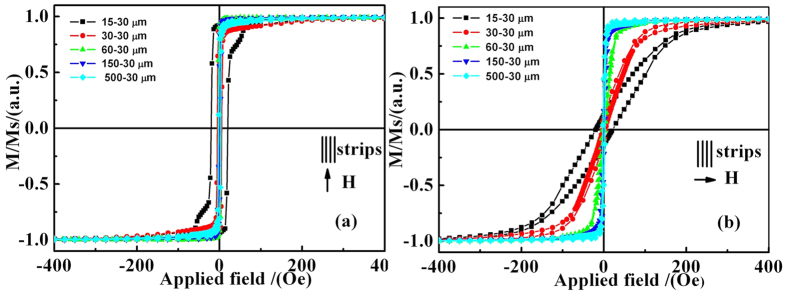
Normalized magnetic hysteresis loops of FeNi strip film as the applied magnetic field: (**a**) paralleled to the long axis of the strip (**b**) perpendicular to the long axis of the strip.

Figure 3 shows a typical normalized magnetic hysteresis loop of the FeNi strip in two directions using vibrating sample magnetometer (VSM). The detailed measuring process is described in the section of Methods. All samples exhibit good soft magnetic properties. All samples have saturation magnetic fields of less than 10 Oe when the applied magnetic field is parallel to the long-axis of the strip as in Fig. 3a. However, when the applied magnetic fields are perpendicular to the long-axis of the strip, the saturation magnetic fields increase gradually from 5 to 300 Oe as *W* decreasing. Samples have very different saturation magnetic fields in different magnetization directions, which means that IPUMA is successfully induced through changing strip widths. To study IPUMA field in more detail, effective *H*_*k*_ are calculated from hysteresis loops and the results are listed in Table 1. *H*_*k*_ increases from 2.2 to 172.4 Oe when the width decreases from 500 to 15 *μm*, so it is a useful method to adjust the IPUMA field by changing *N* which is related to the demagnetization field^20^.

#### Dynamic magnetic properties of the patterned FeNi strip film

**Figure 4:**
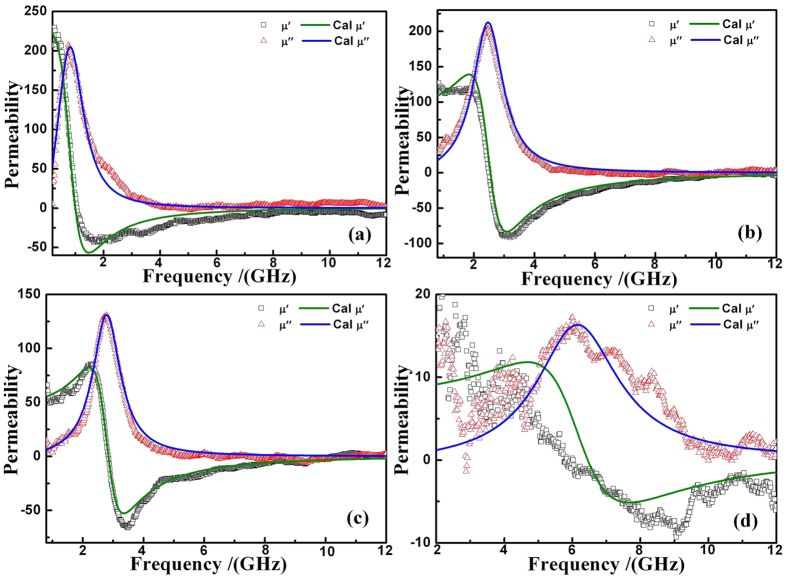
Permeability spectra of FeNi strip films with *d* = 30 *μm* and *W* = 500 (**a**), 60 (**b**), 30 (**c**), and 15 *μm* (**d**).

*f*_*r*_ of the film depends on *M*_*s*_ and *H*_*k*_. The static magnetic properties indicate that the *H*_*k*_ was adjusted successfully. Since the IPUMA field changes with the magnetic strip width, the dynamic magnetic properties of the FeNi strip films can be adjusted by changing the strip width. We investigated the dynamic magnetic properties for frequency ranges from 0.5 to 12 GHz. Figure 4 shows the typical real (*μ*′) and imaginary parts (*μ*′′) of the permeability of the patterned FeNi film with strip widths *W* = 500, 60, 30, and 15 *μm*. The experimental results are fitted to the Laudau-Liftshitz-Gilbert (LLG) equation. The resonance peak of the imaginary parts (*μ*′′) changes from 1 to 6.35 GHz as the *W* decreases from 500 to 15 *μm*. This means that *f*_*r*_ decreases with *W*. Detailed results are given in Table 1. The real part(*μ*′) of the permeabilities are also shown in Fig. 4. *μ*′ decreases with the frequency, which is consistent with the result of 8. *f*_*r*_ of FeNi strip films were adjusted easily by changing strip width.

### FeNi films with different strip width *W* and gap *d* = *W*

#### Static and dynamic magnetic properties of patterned FeNi strip films

The above study indicates the significant influence of *W* to *f*_*r*_. Higher *f*_*r*_ could be obtained for a narrower sample width which has a larger IPUMA field. In this section, We fabricated samples with *W* = *d* = 2, 5, 15, 30 *μm*. The length and thickness of the strips are 5000 *μm* and 120 nm, respectively. A stronger shape anisotropy is expected in this system, which would induce larger *H*_*k*_ and *f*_*r*_.

**Figure 5:**
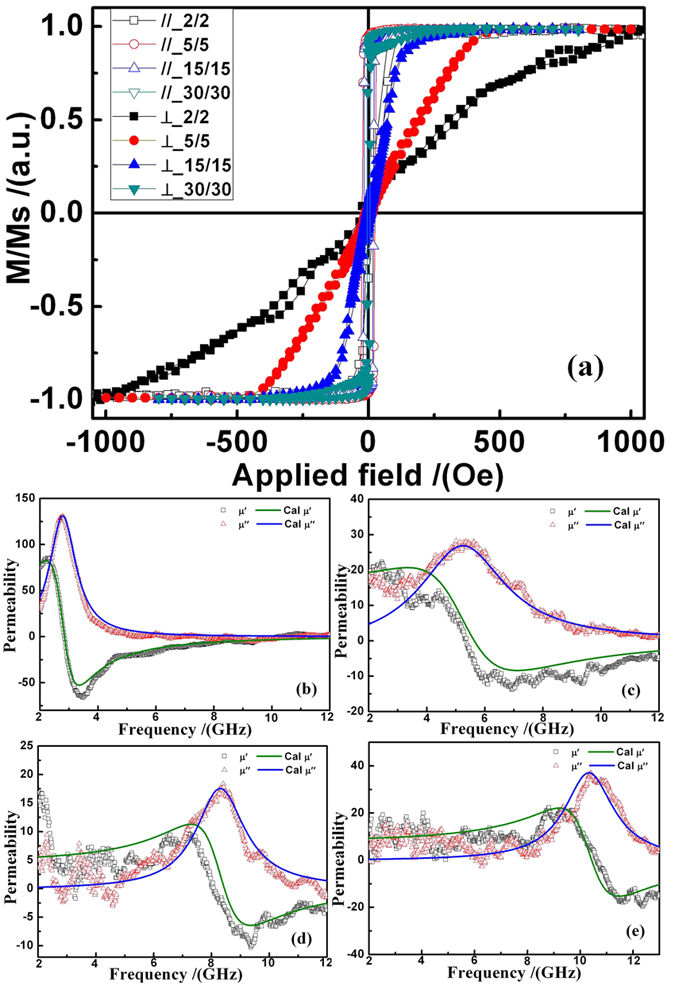
In-plane normalized magnetic hysteresis loops when the applied magnetic field is parallel to (//) and perpendicular to (⊥) the long-axis of strip (**a**) and permeability spectra of FeNi strip film with *W* = *d* = 30 (**b**), 15 (**c**), 5 (**d**), and 2 *μm* (**e**).

Figure 5a shows the in-plane normalized magnetic hysteresis loops of the FeNi strip films. All saturation magnetic fields are also less than 10 Oe when the applied magnetic field is parallel to the long-axis of the strip. When the applied magnetic field is perpendicular to the long-axis of the strip, the magnetic saturation field reaches 1000 Oe for *W* = 2 *μm*. The calculated *H*_*k*_ change from 87.8 to 576 Oe shown in Table 2. A wider range tunable IPUMA field was obtained by decreasing the strip *W* and *d* of the strips. Based on the Kittle equation, a higher *f*_*r*_ of the soft magnetic film can be obtained for a larger IPUMA field. Figure 5b shows the permeability spectra of the four samples for *W* = 30, 15, 5, 2 *μm*. The resonance peaks of *μ*′′ are located at 2.84 GHz, 5.56 GHz, 8.45 GHz and 10.6 GHz for W = 30, 15, 5, and 2 *μm*, respectively (see Table 2). As for the VSM results, *f*_*r*_ changes in the same way with *H*_*k*_, which is due to the shape anisotropy. This indicates that shape anisotropy is crucial to adjust *f*_*r*_ from 2.84 to 10.6 GHz. In comparation with the previous results, an ultra-wide range of resonance frequency shift from 2.84 to 10.6 GHz is obtained by changing IPUMA which is originated from the shape anisotropy in magnetic strips. Wider *f*_*r*_ ranges can be obtained by adjusting the strip width more. This kind of adjustment might induce the shape anisotropy to cooperate or compete with the other anisotropies, such as magnetocrystalline and stress anisotropies.

## Discussion

We found the IPUMA is induced mainly by shape anisotropy which can be changed for different *W*, and *d*. Ultra-large ranges of *f*_*r*_ from 1 to 10.6 GHz have been achieved. We further applied the bi-anisotropy theory to simulate the measured magnetic spectrum^5^. The magnetic resonance frequency can be expressed as 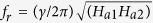, where *H*_*a*1_ and *H*_*a*2_ are the effective anisotropy field when the magnetization deviates from the easy axis in the the easy and hard plane, respectively. So it is convenient to change the *f*_*r*_ by adjusting *H*_*a*1_ and *H*_*a*2_ over a wide range. For the magnetic thin films, *H*_*a*1_ equals to in-plane the effective anisotropy field *H*_*k*_, and *H*_*a*2_ the out-of-plane *H*_*k*_, and there is relation *H*_*a*2_ = *H*_*a*1_ + 4*πM*_*s*_. Thus the ferromagnetic resonance frequency of the patterned strip films can be obtained as:


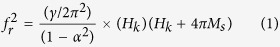


The permeability can be calculated theoretically by the Landau-Lifshitz-Gilbert equation^21^:


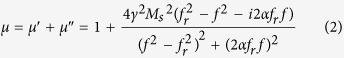


where *α* is the damping factor and, *γ* is gyromagnetic ratio.

**Figure 6:**
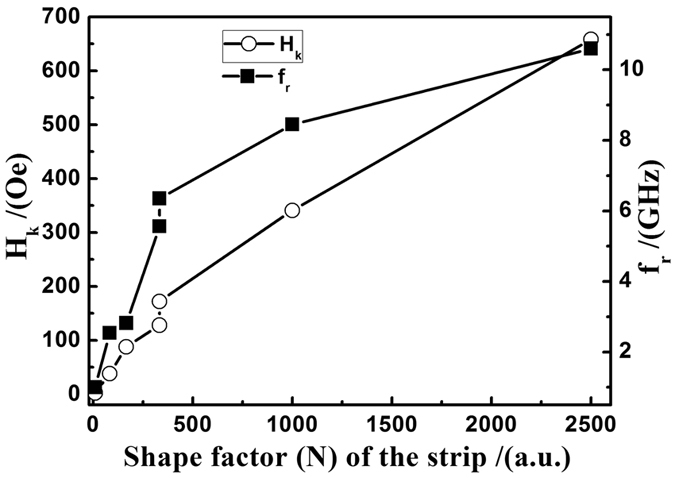
IPUMA field (hollow point) and *f*_*r*_ (solid point) changed with the shape factor (*N* = *L*/*W*).

All experimental data were fitted to Eqs (1) and (2), and the fitting results are given in Figs 4 and 5 (solid line). The damping factors (*α*) of different samples are listed in Tables 1 and 2. *α* changes from 0.1458 to 0.047 as strip shape changes, which is also indicated by the line width of the magnetic spectrum. Figure 6 shows the variation of *H*_*k*_ and *f*_*r*_ with the shape factor *N*. *H*_*k*_ increased from 2.2 Oe to 576 Oe and *f*_*r*_ increased from 1 GHz to 10.6 GHz, the net frequency shift Δ *f*_*r*_ is 9.6 GHz, which, to our best knowledge, might be the widest tuning range for the metal soft magnetic film. *H*_*k*_ and *f*_*r*_ exhibit the same trend, which indicates that an increase in *f*_*r*_ results mainly from shape anisotropy. Different *H*_*k*_ and *f*_*r*_ were observed as *d* changes. For W fixed as 30 *μm* and L 5000 *μm*, the measured resonance frequencies are 5.56 GHz for *d* = 15 *μm* and 6.35 GHz for *d* = 30 *μm*. The main reason is that coupling between strips decreases with *d*, which will lead to the increase of *H*_*k*_. The strip *W* and *d* can be controlled by photolithography process, thus we can fabricate strips of different sizes easily, which means that different shape anisotropies or *H*_*k*_ could be controlled. *f*_*r*_ range from 1 and 10.6 GHz has been achieved, and a wider range might be reached in combination with other anisotropies.

In summary, patterned FeNi strip films were produced by traditional magnetron sputtering and photolithograph. The IPUMA field up to 576 Oe and ferromagnetic *f*_*r*_ from 1 to 10.6 GHz were obtained, which covers four microwave bands of L, S, C and X bands. The magnetic anisotropy strongly depends on the shape anisotropy determined by the *W* and gap *d*. It is a simple method to adjust the static anisotropy and dynamic high frequency properties of the soft magnetic films with or without strong magnetocrystalline or stress. Furthermore, a wider tunable range of the resonance frequency could be obtained if other anisotropies as magnetocrystalline anisotropy, and stress anisotropy are included.

## Methods

### Sample preparation

Patterned Ta/FeNi/Ta film with strip arrays were produced by magnetron sputtering and photolithography method. *Fe*_8_*Ni*_2_ target were used during magnetron sputtering. A photoresist layer was spin coated onto a silicon substrate, then contact optical lithography was applied to fabricate strips with different *W* and *d*. The *W* and *d* varied from 2 to 500 *μm* and 2 to 30 *μm*, respectively. We first deposit Ta/FeNi/Ta films onto the Silicon substrates with patterned photoresist, then we do direct-current magnetron sputtering with a base pressure lower than 1 × 10^−4^ Pa and Ar processing pressure of 0.4 Pa. The thickness of the FeNi layer is 120 nm. The seed and the capping layer are Ta with the same thickness of 5 nm. Patterned FeNi strips are produced by removing photoresist with acetone.

### Measurement

Surface topographies were studied by SEM and AFM methods. The *W*, *d*, and thickness of the patterned Ta/FeNi/Ta films were measured by a Dektak-XT step profiler. The thickness and roughness of the FeNi layer and Ta layer were measured by the Neutron reflectivity method at CMRR. A vibrating sample magnetometer (VSM, BKT-4500Z) was used to study the static magnetic properties of the FeNi films. Permeability spectra of the samples were measured in ranges between 100 MHz and 15 GHz, using the shorted micro-strip transmission-line perturbation method realized by a vector network analyzer (VNA, Agilent PNA8363B). All measurements were performed at room temperature.

## Additional Information

**How to cite this article**: Ren, Y. *et al*. Patterned FeNi soft magnetic strips film with tunable resonance frequency from 1 to 10.6 GHz. *Sci. Rep.* **6**, 31773; doi: 10.1038/srep31773 (2016).

## Figures and Tables

**Table 1:** Width, anisotropy field, *f*_*r*_ and damping factor of the FeNi strip films with fixed *d* = 30 *μm*.

Sample	*W*/(*μm*)	Anisotropy field (*H*_*k*_/Oe)	*f*_*r*_/(GHz)	Damping factor (*α*)
1	500	2.2	1	0.08
2	60	38	2.54	0.047
3	30	87.8	2.84	0.053
4	15	172.4	6.35	0.1435

**Table 2:** Strip width, anisotropy field and *f*_*r*_ of the FeNi strip films with *W* = *d* = 2, 5, 15, 30 *μm*.

Sample	*W or d*/(*μm*)	Anisotropy field (*H*_*k*_/Oe)	*f*_*r*_/(GHz)	Damping factor (*α*)
1	30	87.8	2.84	0.053
2	15	128	5.56	0.1458
3	5	341	8.45	0.09
4	2	576	10.6	0.065
